# Supplementary material for: Derivation of Soil Criteria of Cadmium for Safe Rice Production Applying Soil–Plant Transfer Model and Species Sensitivity Distribution
Source: Int J Environ Res Public Health. 2022 Jul 21;19(14):8854. doi: 10.3390/ijerph19148854 (PMC9315542; doi:10.3390/ijerph19148854)
Supplement: Supplementary file 1 [file ijerph-19-08854-s001.zip › ijerph-1803652-supplementary.pdf]

International Journal of
Environmental Research and Public Health

MDPI

Table S1. The derived soil thresholds of Cd based on soil-plant transfer models.

Protection levels (%)	Soil thresholds			
	pH ≤ 5.5	5.5 < pH ≤ 6.5	6.5 < pH ≤ 7.5	pH > 7.5
95	0.07	0.09	0.11	0.13
90	0.10	0.12	0.15	0.18
80	0.15	0.18	0.22	0.27
50	0.33	0.40	0.49	0.59
20	0.72	0.87	1.07	1.31
10	1.08	1.31	1.61	1.99
5	1.51	1.85	2.27	2.82

The thresholds at soil pH values of 5.0, 6.0, 7.0, and 8.0 were used for scenarios of soil pH ≤ 5.5, 5.5 < pH ≤ 6.5, 6.5 < pH ≤ 7.5, and pH > 7.5, respectively.

International Journal of
Environmental Research and Public Health

MDPI

Table S2. The derived soil thresholds of Cd based on species sensitivity distribution.

**Protection levels (%)**	**Soil thresholds**			
	**pH ≤ 5.5**	**5.5 < pH ≤ 6.5**	**6.5 < pH ≤ 7.5**	**pH > 7.5**
95 (HC_5)	0.06	0.08	0.14	0.09
90 (HC_{10})	0.09	0.13	0.25	0.15
80 (HC_{20})	0.14	0.24	0.50	0.30
50 (HC_{50})	0.35	0.76	1.89	1.03
20 (HC_{80})	1.05	2.43	7.10	3.57
10 (HC_{90})	2.22	4.47	14.18	6.84
5 (HC_{95})	4.63	7.39	25.12	11.70
